# Suppressor of fused (Sufu) represses Gli1 transcription and nuclear accumulation, inhibits glioma cell proliferation, invasion and vasculogenic mimicry, improving glioma chemo-sensitivity and prognosis

**DOI:** 10.18632/oncotarget.2585

**Published:** 2014-10-29

**Authors:** Xing Liu, Xiaofeng Wang, Wenzhong Du, Lingchao Chen, Guangzhi Wang, Yuqiong Cui, Yang Liu, Zhijin Dou, Hongjun Wang, Ping Zhang, Liang Chang, Liye Yi, Jinquan Cai, Chuanlu Jiang

**Affiliations:** ^1^ Department of Neurosurgery, The Second Affiliated Hospital of Harbin Medical University, Harbin, China; ^2^ Department of Neurosurgery, Huashan Hospital, Fudan University, Shanghai, China; ^3^ Chinese Glioma Cooperation Group (CGCG)

**Keywords:** glioma, Sufu, Gli1, temozolomide, hedgehog

## Abstract

Glioblastoma are highly aggressive brain tumors with poor prognosis. While various dysregulation of signaling pathways in gliomas have been described, the identification of biomarkers and therapy targets remains an important task for novel diagnostic and therapeutic approaches. Here we described that the Suppressor of fused (also known as Sufu) is significantly down-regulated in high-grade gliomas, correlating with a poor prognosis. We demonstrated that ectopic expression of Sufu inhibited cell proliferation, invasion and vasculogenic mimicry. In addition, overexpression of Sufu reduced Gli reporter gene transcription activity and prevented Gli1 nuclear accumulation, whereas knockdown of Sufu reversed these effects. Furthermore, overexpressed Sufu sensitized glioblastoma to Temozolomide and Cyclopamine. Thus, Sufu is potential tumor suppressor and therapeutic target in glioblastoma.

Malignant primary brain tumors are major causes of cancer-related mortality in children and young adults [1]. Glioblastoma (GBM), which is the most common and lethal type of primary brain tumor, is highly infiltrative, rapidly progress and demonstrating relative resistance to both radiotherapy and most chemotherapeutic agents. The median survival of GBM is about 12–15 months despite aggressive comprehensive treatment [2–4]. Although clinical and pathological subtype study of GBM has been increasing in recent years, there are still few prognostic markers and predictors of therapeutic response yet [5, 6].

Suppressor of fused (Sufu) is a powerful negative regulator of hedgehog, WNT and other signaling pathways in vertebrates and prevents development of malignant tumors [7–10]. Loss of Sufu function promotes tumorigenesis. In gastric cancer, reduced Sufu expression were found to be typical events in tumor tissues [11]. In basal cell carcinoma (BCC), Sufu restricts the activity of Gli2 through cytoplasmic sequestration and Sufu/kif7 simultaneous deletion induces BCC [12]. Furthermore, mutation of Sufu was associated with familial multiple meningioma, medulloblastoma and Gorlin syndrome [13–15]. Particularly, researchers revealed that Sufu^−/−^ animals developed medulloblastoma and rhabdomyosarcoma in conjunction with p53 loss and the following hedgehog antagonist treatment did not block growth of tumors arising from Sufu inactivation [7]. These researches demonstrate that Sufu is essential for individuals' development and functions as a tumor suppressor. Nonetheless, differential expression, intracellular function and latent mechanism of Sufu in human glioma have not yet been investigated.

In this study, we analyzed Chinese Glioma Genome Atlas (CGGA) and observed that Sufu expression was an independent risk factor for glioma patients overall survival (OS). Further, we indicated that ectopic expression of Sufu suppresses glioma cell growth, invasiveness, and angiogenesis through Hedgehog signaling pathway via Gli1 directly binding and subcellular distribution both *in vitro* and *in vivo*. Moreover, Temozolomide and Cyclopamine efficiency is enhanced by exogenous Sufu expression, which could benefit the clinical treatment for glioma patients.

## RESULTS

### The expression of Sufu reduced along with grade progression

**Figure 1:**
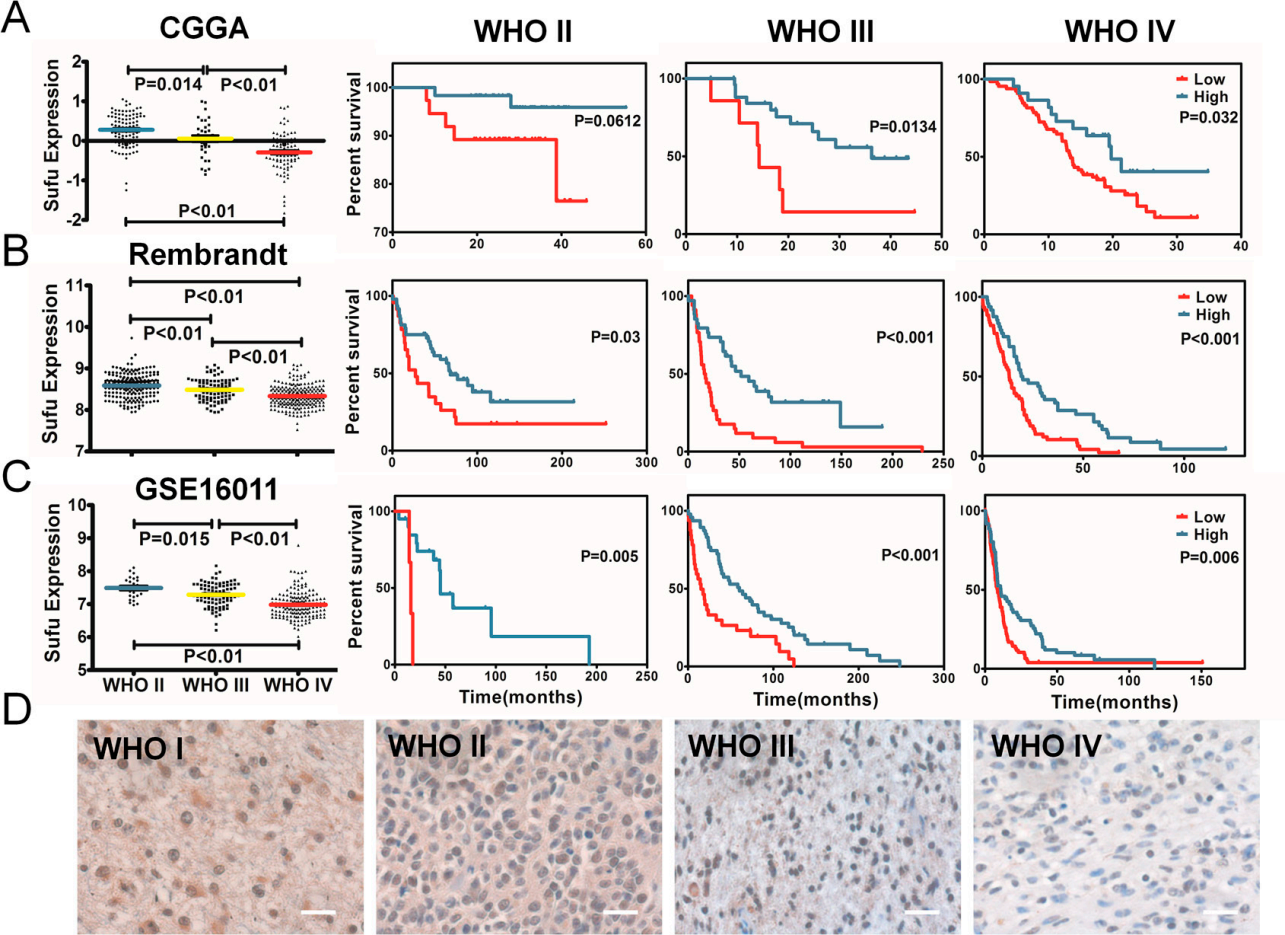
The expression difference and prognostic value of Sufu in glioma tissues **(A)** the expression difference of Sufu and correlationship with glioma grade prognosis in CGGA. A single spot was the expression value of Sufu of an individual patient. Lines in the middle were the mean expression value. According to Sufu expression level, patients with every grade could be divided into two groups with significantly different prognosis, respectively. **(B) (C)** the same conclusion was observed from other two validation datasets (Rembrandt and GSE16011 data). **(D)** Immunohistochemical staining showed Sufu expression reduced along with grade progression of gliomas.

Through analyzing the discovery sets, we found that Sufu was differently expressed in all grades of gliomas significantly. Sufu expression reduced accompanied by grade progression of glioma both in CGGA and the other two validation datasets (Figure 1). The expression status of Sufu was further detected in 30 glioma patients by IHC. Similar to datasets analysis, IHC of 30 glioma patients indicated that Sufu was lower in high grade gliomas (WHO III, WHO IV) than that in low grade gliomas (WHO I, WHO II) (*P* < 0.01) (Figure 1D). Then, we confirmed the prognosis of CGGA patients. The optimal cutoff for comparison of OS was identified in a randomly split cohort. After elimination of patients with unavailable or too short OS, remaining patients went for further prognosis analysis. Results showed that glioma patients with high expression of Sufu had favorable prognosis in CGGA (except WHO II, *P* = 0.0612) (Figure 1A, *P* < 0.05), testified by the other two validation datasets (Figure 1B, 1C) and TCGA (Supplementary Figure 2). Thus, Sufu was a prognostic marker in every grade gliomas.

TCGA network describes a robust gene expression-based molecular classification of GBM into proneural, neural, classical, and mesenchymal subtypes, whereas CGGA derived 3 major groups called G1, G2, and G3 [3, 16]. To explore which subtype high- or low-level of Sufu belongs to, we analyzed CGGA database and divided patients into two parts according to median. As shown in Table 1, patients with high level of Sufu are younger, low malignancy, IDH1 mutant and mainly belong to G1 and Proneural subtype. Further, the other patients are elder, high WHO grade, IDH1 wild type and mainly G3 and Mesenchymal subtype.

**Table 1:** Clinical features of patients with different expression of Sufu in CGGA database

Varible		Sufu-High (n = 110)	Sufu-Low (n = 110)	*P* value
**Median Age**		38	45	
**Age**	≥45	34	58	
	<45	76	52	0.001
**Gender**	Male	50	46	
	Female	60	64	0.628
**Preoperative KPS score**	≥80	92	82	0.097
	<80	18	28	
**Grade**	WHO II	71	26	<0.001
	WHO III	18	16	
	WHO IV	21	68	
**IDH1 status**	Mutation	68	18	<0.001
	Wild type	23	78	
	NA	19	14	
**CGGA Subtype**	G1	41	1	<0.001
	G2	43	39	
	G3	26	70	
**TCGA Subtype**	Classical	19	19	<0.001
	Mesenchymal	26	50	
	Neural	20	35	
	Proneural	45	6	

### Sufu inhibited proliferation, restrained migration and vasculogenic mimicry of glioma cells

U87, U251 glioma cell lines were selected as Sufu low-expression and high-expression cell lines used in subsequent experiments after mRNA and protein detection (Supplementary Figure 3).

**Figure 2:**
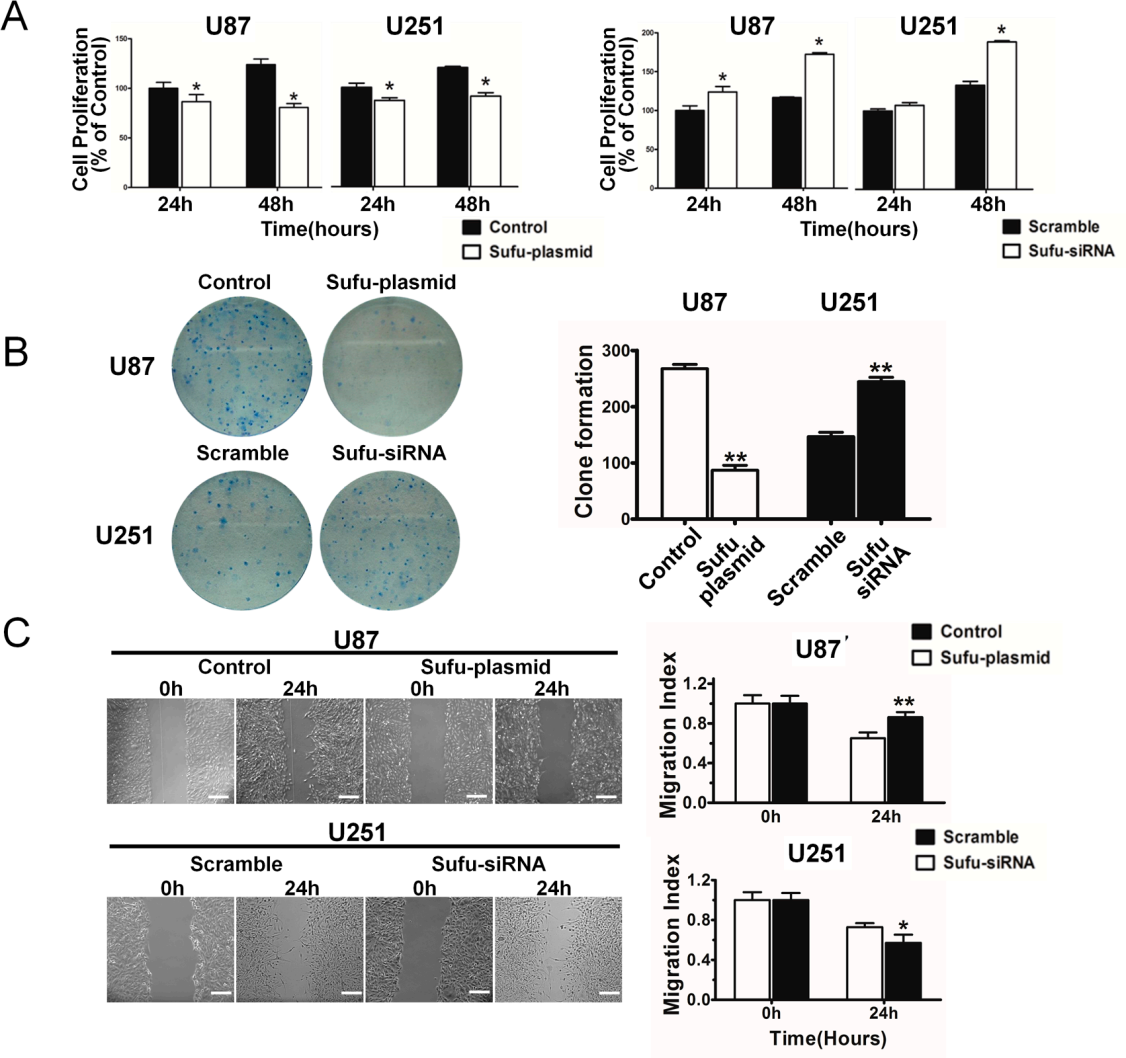
Sufu inhibited proliferation and restrained migration of glioma cells **(A)** Cell viability was examined with MTT assay in different time intervals after transgene expression. **(B)** Histogram and images showing the total numbers of colonies formed after transgene expression. **(C)** For wound healing assay, the scratch was photographed at 0 h and after 24 h after transfection. The area between cells was calculated, standardized against control or scramble (normalized to control). Bars represent 250 μm. Data represent mean±SEM of three replicates. *P < 0.05; **P < 0.01.

**Figure 3:**
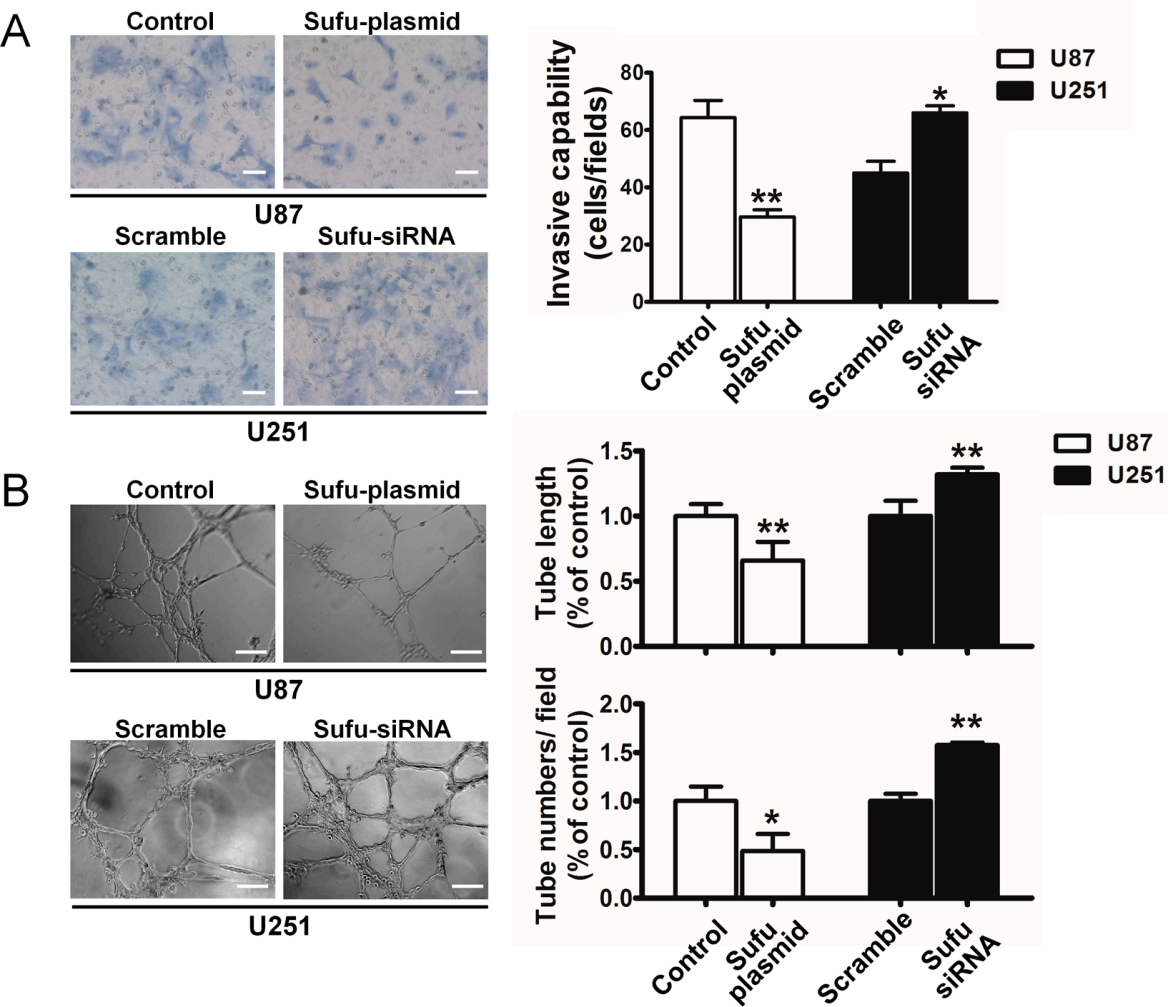
Sufu suppressed invasion and vasculogenic mimicry of glioma cells **(A)** Representative images of transwell assays of U87 and U251 after transfection. Number of invaded cells shown as a histogram. Bars represent 50 μm **(B)** Representative images of VM assay tube structures. Both tube number/field and tube length were measured. Bars represent 100 μm. Data represent mean ± SEM of three replicates. *P < 0.05; **P < 0.01.

To examine the effect of Sufu on proliferation, MTT assay was performed (Figure 2A). The results showed a striking reduce in the growth and indicated that cell proliferation was inhibited *in vitro* after transfection with Sufu plasmid or plain vector in U87, U251 cells in different time points (24, 48h) (*P* < 0.05). Simultaneously, Sufu siRNA or control siRNA enhanced cell proliferation significantly (Figure 2A) (*P* < 0.05). Moreover, the colony formation was suppressed by Sufu elevating and increased by Sufu silencing (Figure 2B) (*P* < 0.01). Malignant glioma cell lines presented highly invasive growth characteristics *in vitro* and *vivo*. Thus, wound-healing and transwell invasion assay were performed to test whether the Sufu could influence cell migration and invasion *in vitro*. Results displayed that Sufu downregulation enhanced migration and invasion of U251 cells compared with control (Figure 2C, 3A). Conversely, Sufu upregulation attenuated migration and invasion of U87 cells (Figure 2C, 3A). Brain tumor growth and progression depends on the establishment vascular, which confers a tremendous survival and growth advantage on the malignant cells [17]. Among various types of vascularization, vessels formed by tumor cells (vasculogenic mimicry, VM) provided a complementation to tumor's blood supplement [18] and correlated with increasing malignancy and higher aggressiveness of glioma [19]. For this reason, we evaluated Sufu effects on tumor angiogenesis using glioma cells vascular mimicry assay. As shown in Figure 3B, both tube length and tube number/filed were significantly decreased in Sufu-overexpressed group compared with negative control (*P* < 0.05), whereas knockdown of Sufu induced more tube-like structures (Figure 3B, *P* < 0.01). To further understand Sufu effects on tumorigenesis, we selected Human Astrocytes (HA), the normal brain cells to proceed following trials. The knockdown efficiency of Sufu siRNA on HA was tested by WB (Supplementary Figure 4B). Then, MTT and EdU proliferation assay was performed. According to the results, the growth of HA with low expression of Sufu was more prosperous than those of high Sufu level (Supplementary Figure 4A, C). Collectively, our results indicate that Sufu regulates cell proliferation, migration and angiogenic ability of glioma cells *in vitro*.

### Sufu bound to Gli1 and repressed Gli1 transcription activity

**Figure 4:**
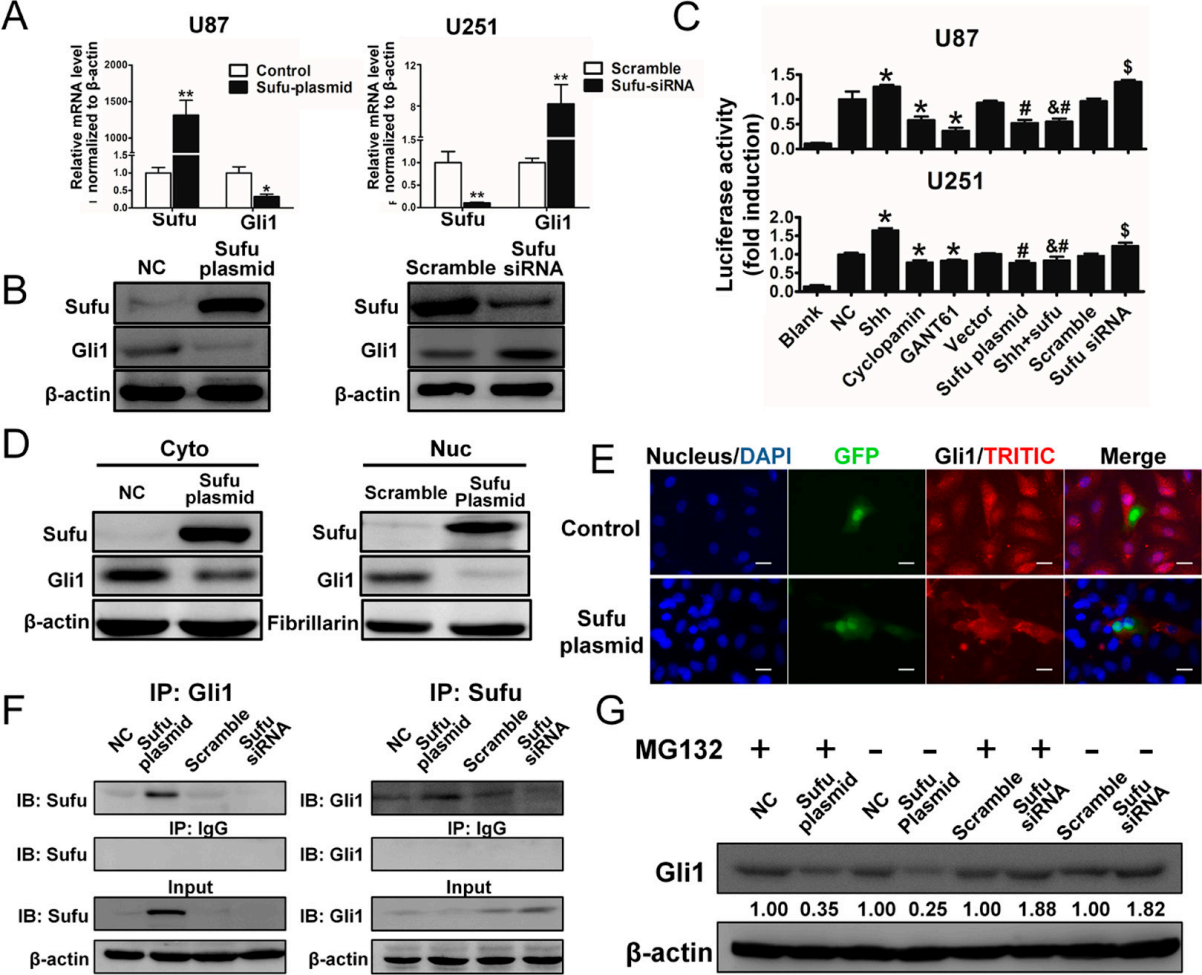
Sufu suppressed Gli1 transcription activity via directly bound to Gli1, resulting Gli1 nuclear-localization inhibition and proteolysis **(A, B)** mRNA and protein expression changes after Sufu elevating in U87 cells and silencing in U251 cells. *P < 0.05; **P < 0.01. **(C)** U87 and U251 cells were transiently transfected (24 h) with an 8x-Gli1 plasmid and co-transfected with pGL4.75. Cells were then treated as indicated. Both firefly and Renilla luciferase activities were quantified and the firefly/Renilla luciferase activities were recorded as fold induction. Mean ± SEM (n = 3). *P < 0.05 to NC group, #*P* < 0.05 to Vector group, &*P* < 0.05 to recombinant Shh group and $*P* < 0.05 to scramble group. **(D)** Sufu effects on Gli1 nuclear localization verified by WB assay with cytoplasmic and nuclear proteins. **(E)** Representative images of IF using anti-Gli1 antibody shows the subcellular distribution of Gli1 protein. Cells transfected with Sufu or plain plasmid were confirmed by its GFP protein expression. Bars represent 50 μm. **(F)** Co-IP assay to investigate interaction between Sufu and Gli1. Sufu plasmid and siRNA were used to confirmed effects of different Sufu expression status on Sufu-Gli1 association. (G) Transfected cells treated with MG132 (10 μM) for 6 h and lysed for WB analysis. Data represent mean ± SEM of three replicates.

Above results indicated that Sufu is a prognosis marker in glioma and reverse glioma malignance. However, the underlying mechanism was poorly understood and deserved more investigation. Former studies revealed that Gli1, a transcription factor of hedgehog signaling pathway, is a direct target of Sufu and is essential for tumorigenesis [20–22]. Therefore, to investigate how Sufu influence glioma cells, we firstly detected Gli1 and Sufu mRNA expression changes after transfection. Results indicated that in U87 cells, Gli1 mRNA was significantly down-regulated when Sufu expression elevated (*P* < 0.05). Similarly, silencing Sufu by siRNA significantly induced Gli1 mRNA expression in U251 (Figure 4A). WB analysis further verified Sufu effect on Gli1 expression (Figure 4B). We next confirmed the association between Sufu and Gli1 through a co-IP assay in U87 cells using anti-Sufu (or anti-Gli1) antibody or control rabbit IgG followed by immunoblotting for Gli1 (or Sufu). Sufu could bind to Gli1 directly and the association of Sufu-Gli1 could be enhanced by exogenous expression of Sufu and weaken by knockdown of endogenous Sufu (Fig. 4F). Moreover, after treatment of recombinant Shh (rShh, 1 μg/mL, R&D), GANT61 (30 μM, Sigma-Aldridge), Cyclopamine (10 μM, Sigma-Aldridge), Sufu plasmid or plain vector and Sufu siRNA or control siRNA for another 48 h, we found that GANT61, Cyclopamine and Sufu plasmid transfection repressed luciferase activity significantly compared with control or vector group, meanwhile rShh, Sufu siRNA greatly enhanced the activity compared with control or scramble group (Figure 4C) (*P* < 0.05). These data revealed that Sufu suppressed Gli1 activation. Impressively, overexpression of Sufu reduced the expression of Gli1 induced by hedgehog signaling agonist rShh.

### Sufu inhibited Gli1 nuclear localization and promoted Gli1 proteolysis

Gli1 functions as a transcription factor by trans-locating to nucleus, where it binds to a consensus Gli1-binding element in target genes resulting in their activation [23]. For this reason, nuclear accumulation is critical for Gli1 functions. Hence, we used IF and WB analysis of cytoplasmic and nuclear protein level to confirm Sufu inhibition on Gli1 expression in U87 cells. In Figure 4E, Sufu transfected cells showed reduced accumulation of Gli1 compared to vector control group. Further, in Figure 4D, cells with high expression of Sufu suppressed Gli1 nuclear localization tested by WB. Moreover, Sufu suppressive effects on Gli1 protein were weaken after treated with 10 μM of the protease inhibitor Carbobenzoxyl-leucinyl-leucinyl-leucinal-H (MG132, Selleckchem) for 6h, suggesting that Sufu could inhibited Gli1 by proteolysis (Figure 4G).

### Expression of Sufu correlated with glioma cells sensitivity to Temozolomide and Cyclopamine

Clinical trials have shown that patient survival can be extended by additional Temozolomide (TMZ) therapy [24]. Even so, resistant of glioma to TMZ restricted its clinical effectiveness. To explore whether different status of Sufu level effect TMZ therapy, we performed MTT assay. In Supplementary Figure 5A, U87 cells transfected with Sufu plasmid was more sensitive than control, while knockdown of Sufu by siRNA increased U87 TMZ resistance. To investigate the mechanism, we analyzed CGGA to determine the relationship of Sufu and O^6^-methylguanine-DNA methyltransferase (MGMT), a methyltransferase which can reverse TMZ-induced cytotoxicity [25, 26]. Results demonstrated that Sufu and MGMT expression was negatively correlated (Supplementary Figure 5B) (R = −0.2655, *P* = 0.014). Same relationship was testified by qRT-PCR (Supplementary Figure 5C) that Sufu mediates MGMT expression, which partially illustrated correlationship between Sufu and TMZ therapy.

Cyclopamine, produced by hellebore, could inhibit proliferation and induces apoptosis in cancer cells by targeting the key oncogene Smo of Hedgehog pathway [27]. In our study, we testified the effect of Cyclopamine on glioma cell lines, either (Supplementary Figure 5D). 5 μM and 10 μM Cyclopamine both repressed U87 cell viability strongly. Further, over-expressed Sufu combined with 5 μM Cyclopamine could suppressed glioma cells proliferation significantly compared with Cyclopamine alone (Supplementary Figure 4E). This may provide a new approach for clinical glioma treatment, especially the Cyclopamine-resistant patients.

### Sufu inhibited tumor growth *in vivo* and prolonged the survival period

**Figure 5:**
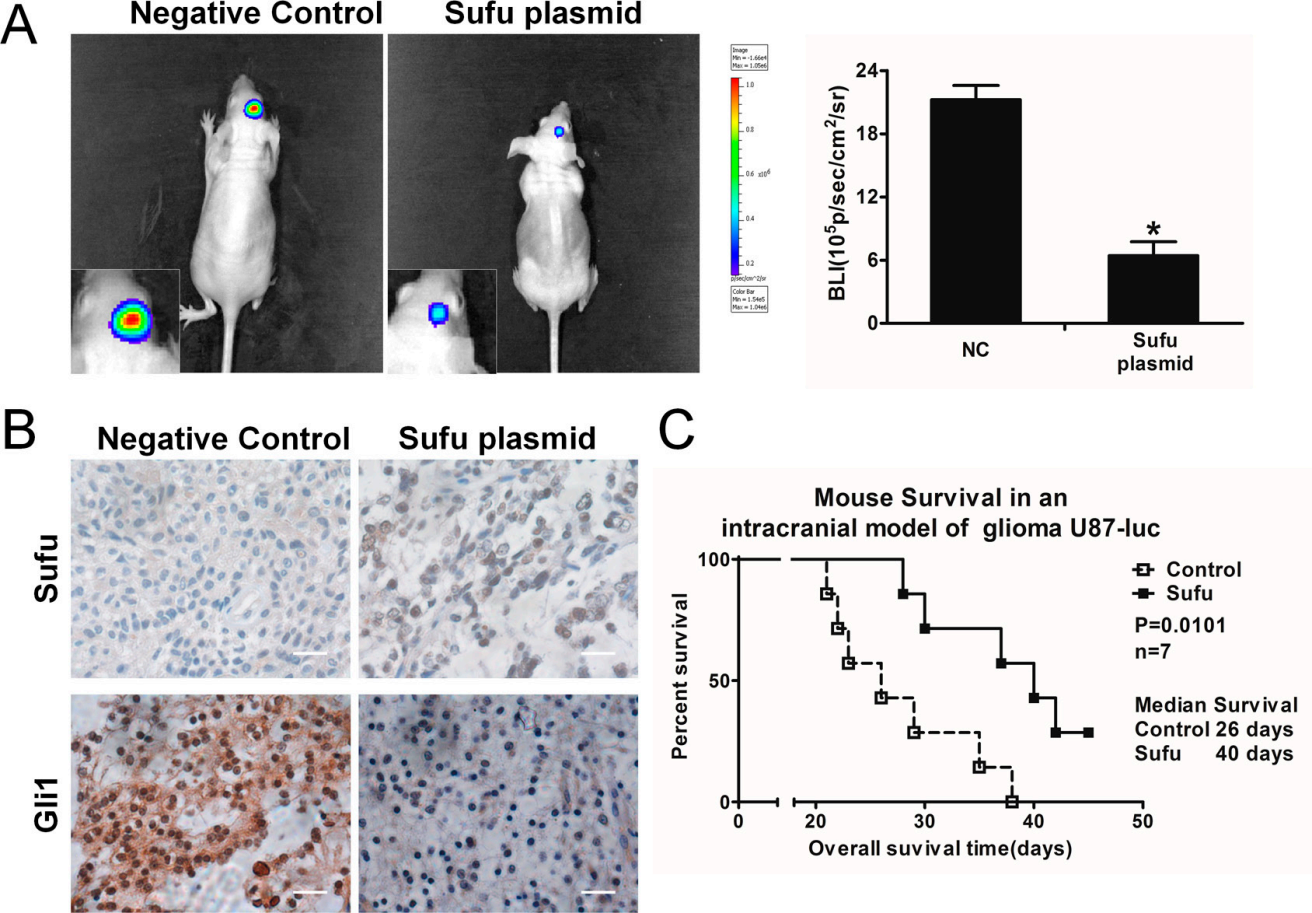
Sufu inhibited glioma *in vivo* growth and prolonged survival time **(A)** Luminescence imaging for Sufu-transfected U87-luc tumors versus controls group. **(B)** Sufu and Gli1 expression in tumor sections following IHC analysis. **(C)** Kaplan–Meier survival curves of mice with the Sufu or plain vector transfection. *P < 0.05; **P < 0.01. Data represent mean ± SEM of three replicates.

Because ectopic expression of Sufu suppressed invasion, proliferation and angiogenesis of glioma cells *in vitro*, we further assessed its effect *in vivo*. When the mice were intracranially transplanted with U87-luc cells that stably express Sufu or plain vector, bioluminescence imaging was done for the whole mice. Sufu-treated U87 cells displayed a considerable reduction of tumor (Figure 5A). IHC analysis confirmed that Sufu plasmid enhanced Sufu expression and reduced Gli1 expression and nuclear accumulation in tumor, compared with control group (Figure 5B). To better evaluate the effect of Sufu on nude mice survival, the survival period of each group (n = 7/group) was analyzed by Kaplan–Meier curve. The Sufu overexpression group showed a significant improvement in survival until the end of the observation period compared to the plain vector controls (*P* = 0.0101) (Figure 5C). Accordingly, results demonstrated that Sufu functions similarly *in vivo*.

### Sufu expression inversely correlated with Gli1 in human glioma and acted as an independent prognostic factor

**Figure 6:**
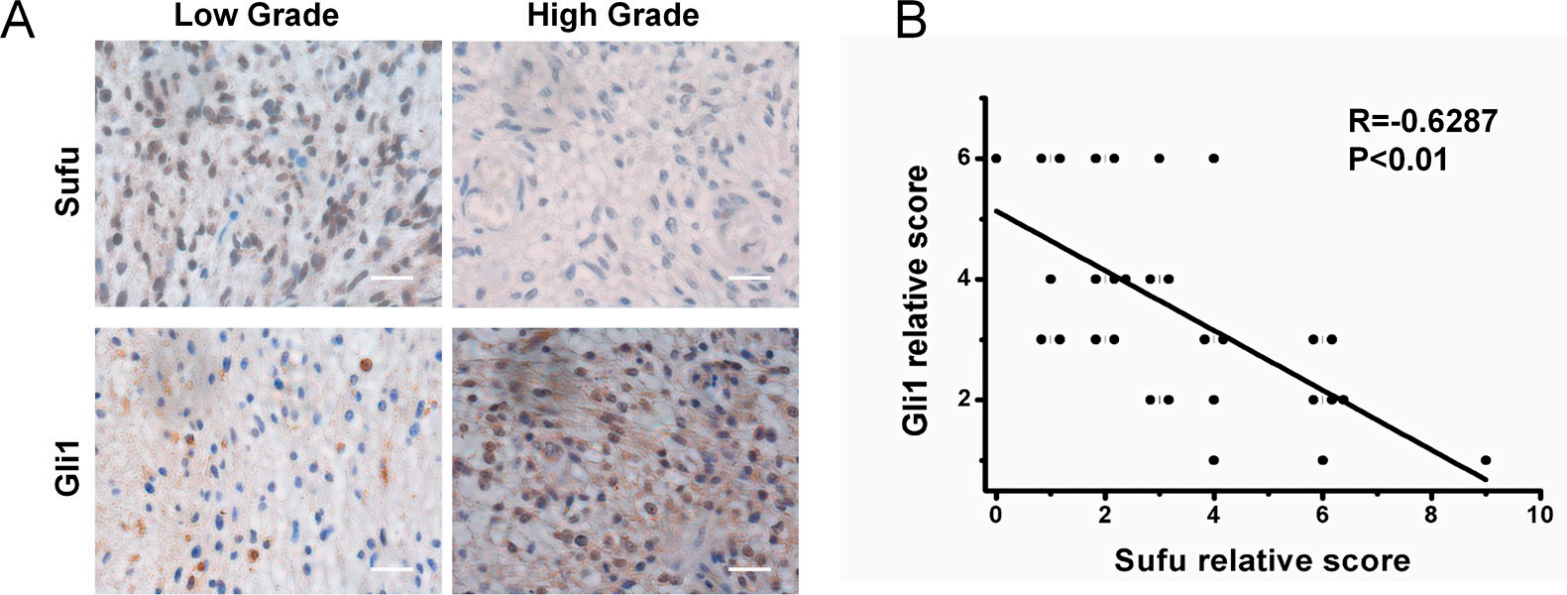
Sufu was negatively correlated with Gli1 expression in human glioma tissues **(A)** Expression of Sufu and Gli1 in resected glioma specimen was assessed by IHC assay. **(B)** A statistically significant negative correlation of Sufu and Gli1 scores in resected glioma specimens (Spearman's correlation analysis, R = −0.6287, *P* < 0.01).

To determine whether the inverse relationship between Sufu and Gli1 expression was consistent *in vitro* and in patient samples, we quantified expression levels of Sufu and Gli1 in glioma tissue specimens by IHC assay and datasets analysis. High-grade glioma contained comparatively lower Sufu expression and higher Gli1 (Figure 6A). Spearman's correlation analysis of IHC demonstrated that Sufu in tumor tissues inversely correlated with Gli1 expression (Figure 6B). Similarly, Pearson's correlation analysis of CGGA revealed that the relationship between Sufu and Gli1 was negatively (Supplementary Figure 6). In 58 WHO IV glioma patients received standard treatment and selected from CGGA database, Sufu expression was correlated with OS by univariate analysis (*P* = 0.017) (Table 2). The median OS time of Sufu high-expressed patients was 408 days, while it was 319 days for low Sufu expression patients. Karnofsky performance status (KPS) score was correlated with OS (*P* = 0.008), either. There were no significant associations between age, gender and OS. The multivariate Cox proportional hazards model identified Sufu expression as an independent prognostic factor for OS (Relative Risk, 2.041; *P* = 0.019). In summary, these data indicate that Sufu has significant clinical impact on glioma.

**Table 2:** Variables related to OS in 58 high-grade glioma with combined treatment: univariate and multivariate analysis

Variable	No. of patients	Univariate analysis				Multivariate analysis		
		Median OS (days)	95% CI(days)	*P*-value		Relative risk	95% CI	*P*-value
**Gender**								
Male	35	325	296–351					
Female	23	396	305–426	0.873				
**Age (years)**								
≤50	33	399	253–373					
>50	25	325	306–416	0.262				
**KPS**								
<80	25	253	274–280					
≥80	33	396	334–442	0.008		2.459	1.396–4.331	0.002
**SUFU-expression**								
Low	40	319	266–359					
High	18	408	328–476	0.017		2.041	1.124–3.706	0.019

CI, confidence interval; KPS, Karnofsky performance status.

## DISCUSSION

The Suppressor of fused (Sufu) is an essential negative regulator for mammals and plays vital roles in multi-biological process. Loss of Sufu is associated with tumorigenicity reported in different cancers. Philip J. Stephens, et al. undertook exome sequencing in a series of 24 adenoid cystic carcinomas (ACCs) and identified that Sufu is one of multiple mutated genes, which could shed light on the molecular underpinnings of ACCs [28]. Further, a subset of medulloblastoma patients carried germline and somatic Sufu mutations suggesting Sufu as a tumor suppressor [15]. Reduced Sufu expression or Sufu mutations were also found to be typical events in other tumor tissues, including gastric cancer, familial multiple meningioma, Gorlin syndrome, basal cell carcinoma and rhabdomyosarcoma. However, there have been no reports investigating the role of Sufu and its molecular mechanism in glioma yet. In present study, we showed that Sufu expression is down-regulated along with glioma grades and there was a negative correlation between Sufu expression and advanced clinical pathological features. To explain this phenomenon, we analyzed Sufu sequence through UCSC Genome Browser on Human (http://genome.ucsc.edu) and confirmed the existence of CpG islands near transcription start sites, which is deactivated under normal state. CpG islands of Sufu could be methylated during glioma formation and lead to gene inactivity, which will result in the loss of Sufu function. What's more, professor Yang and his colleagues advocated that Sufu was a potential target of miR-378 and James G Patton, et al. demonstrated that miR-214 could repress the expression of Sufu, either [29, 30]. Therefore, the regulatory function of microRNA (miRNA) may be another potential mechanism.

The G1 subgroup was characterized by good clinical outcome, young age, low malignant behaviors, and extraordinary high IDH1 mutation [16]. And one of the major features of the Proneural class was point mutations in IDH1 [3]. Notably, the patients with high expression of Sufu were younger, higher rate of mutations in the IDH1 gene and better outcome, either. These indicated that high level of Sufu belongs to G1/Proneural class and associated with favorable prognosis.

As reported previously, infiltrative invasion, incontrollable proliferation and excessive vascularization are hallmarks of glioblastoma multiforme (GBM) [31–33]. In our study, functional experiments via gain- or loss-of-function studies showed that Sufu overexpression suppressed glioma cell proliferation, invasiveness, vascular mimicry (VM) and *in vivo* tumor growth. Conversely, Sufu silencing with siRNA increased these effects. Hedgehog signaling pathway is a morphogen, which determines cell fate during development [27]. Activation of glioma-associated oncogene homolog 1 (Gli1), which was a nuclear transcription factor overexpressed in human glioma [34], enhanced the expression of multiple important Gli1 directly regulated oncoproteins significantly, including CyclinD1/D2 [35], Foxm1 [36], Bcl-2 [37], and epithelial-to-mesenchymal transition (EMT) related proteins [38]. Meanwhile, studies indicated MMP2/MMP9, MT1-MMP, and VEGF could also be mediated by Gli1 activation, inevitably lead to invasiveness and vascularization differences [37, 39]. Inhibition of Gli1 induces DNA damage, cell cycle arrest, cell apoptosis and tumor metastasis [40]. Our results demonstrated that Sufu combined with Gli1 directly to form Sufu-Gli1 complex, which could inhibit Gli1 nuclear accumulation and sequentially repressed Gli1 transcription activity. Meanwhile, the complex is transported to proteasome and degraded. Accordingly, the latent mechanism of Sufu effects on glioma biological behavior could be owed to the inhibition of Gli1 transcription and suppression of downstream genes.

Nowadays, temozolomide (TMZ) has become a key therapeutic agent for patients with malignant gliomas multimodality treatment. However, recent studies observed the resistance to TMZ in malignant gliomas, which attenuated its survival benefit. In our study, combined therapy of TMZ and exogenous Sufu expression significantly suppressed glioma cell proliferation than TMZ group. According to previous researches, O6-methylguanine-DNA methyltransferase (MGMT), which causes the replication of DNA and the growth of malignant gliomas via removal of the alkylating lesion at the O6 position of guanine, may partially contribute to glioma resistance to TMZ therapy [41, 42]. By analyzing mRNA expression of CGGA and qRT-PCR, we observed that Sufu expression was negatively correlated with MGMT expression and Sufu elevation repressed MGMT expression, suggesting that Sufu enhanced TMZ sensitivity by down-regulating MGMT transcription, which further emphasizes the clinical significance of Sufu. Moreover, isocitrate dehydrogenase (IDH) mutation is regarded as a founder biochemical event in tumorigenesis that triggers methylation of MGMT gene [43, 44], which leads to increased sensitivity of TMZ therapy. Thus, to further investigate the latent mechanism of Sufu effects on TMZ treatment, its relationship with IDH mutation deserved more attention. Cyclopamine is a naturally occurring steroidal alkaloid that attenuates the Hedgehog signaling pathway by inhibiting the Smo receptor [27]. Recently, multi-target therapy was introduced for clinical trials and more effective than single target drugs. Here, we demonstrated that combination chemotherapy – incorporating a Smo inhibitor and Gli1 suppressor – could benefit glioma patients of traditional drug resistance, especially those resistant to Cyclopamine.

In conclusion, our study demonstrated that Sufu reduced along with glioma grade and is an independent prognostic factor for glioma patients, suggesting that it could be used as a prognostic biomarker for outcome and may represent a future therapeutic target.

## MATERIALS AND METHODS

### Ethics statement

Informed consent was obtained from all patients involved in this study, and the study protocol was approved by the Clinical Research Ethics Committee of the 2^nd^ affiliated hospital of Harbin Medical University. The protocol for all animal studies was approved by the Clinical Research Ethics Committee of the 2^nd^ affiliated hospital of Harbin Medical College, either.

### Tissue samples and clinical datasets

Samples were consisted of 12 World Health Organization (WHO) grade I-II tumors and 18 WHO grade III-IV tumors (Supplementary Table S1). Freshly resected tissue samples were immediately frozen in liquid nitrogen for subsequent experiments. 220 glioma samples of all grades from Chinese Glioma Genome Atlas (CGGA) database [16] were obtained as discovery set and the Cancer Genome Atlas (TCGA) database [3], the Repository for Molecular Brain Neoplasia Data (REMBRANDT) and GSE16011 data [45] were obtained as validation sets.

### Plasmids and small interfering RNA (siRNA)

Sufu (NM_016169) plasmid and plain vector (CMV-MCS-EGFP-SV40-Neomycin) were purchased from Genechem Company (Shanghai, China). Sufu siRNA and Control siRNA were obtained from Ribobio Company (Guangzhou, China). The Sufu siRNA1, siRNA2 and siRNA3 target sequence were listed in Supplementary Table S2 and knockdown efficiency were testified by qRT-PCR (Supplementary Figure 1).

### Cell Lines and culture condition

Human glioma cell lines (U87, U251) were purchased from Chinese Academy of Sciences Cell Bank. All cells were cultured in DMEM supplemented with 10% fetal bovine serum (FBS, Bioind) and 1% antibiotic (Gibco) at 37°C in a humidified atmosphere with 5% CO_2_ and 95% air. The cells were passaged every 2 days. Stable U87 cell line for the overexpression of Sufu or plain vector were selected using 0.8 mg/mL G418 (Amresco) for 2 weeks and then cultured in 10% FBS with 0.4 mg/mL G418.

### Cell proliferation assay and colony formation assay

A total of 2000 exponential phase cells were plated onto each well of 96-well plates (100 μL medium/well) and cultured overnight. Then, U87 and U251 cells were transfected with Sufu plasmid or plain vector, siRNA or control siRNA for another 48 h. At different time interval, 10 μL/well MTT solution (Sigma-Aldrich) was added and incubated for 4 h. Subsequently, the medium was replaced by DMSO for 10 min and quantified formazan amount with IMARK microplate reader at 490 nm of absorbance. For colony formation assay, 300 transfected cells were seeded in 6-well plates and cultured for another 12 days in complete medium until megascopic colonies appeared. The colonies obtained were washed with PBS and fixed in 10% formaldehyde for 10 min and stained with Giemsa stain at room temperature. Each experiment was performed in triplicate.

### Wound healing assay and transwell invasion assay

U87 and U251 cells were seeded in 6-well plates and cultured until confluency. Then, cells were transfected with plasmids or siRNA and a scratch was created by manually scraping the cell monolayer with a 200-microliter sterile pipette tip. The cells were washed twice with PBS and then incubated in DMEM without FBS. Photographs of the scratched area were taken at 0 h and after 24 h using an Axiovert 200 microscope (Carl Zeiss) and analyzed by Image pro-plus software. The scratch was captured in six different photographs.

The transwell invasion assay was done in 24-well cell culture chambers using transwell inserts (Corning) with 8-μm pore membrane pre-coated with Matrigel (BD Bioscience) [46]. Briefly, transfected cells were plated at the density of 1 × 10^4^ per upper well in 200 μL culture medium (DMEM only). The lower chamber was filled with 500 μL medium (DMEM, 12% FBS). 24 h later, upper surface were removed by scrubbing with a cotton-tipped swab, while lower surface were fixed for 30 min in methanol, air-dried briefly and stained with crystal violet. All experiments were performed in triplicate.

### Vascular mimicry assay

Vascular mimicry assay was processed as described previously [47]. In sum, 3 × 10^4^ transfected U87 and U251 cells were seeded onto 48-well pre-coated with Matrigel. Tube formation was assessed under a phase-contrast microscope 8 h after seeding. All experiments were performed in triplicate.

### Quantitative real-time PCR

Total RNA was extracted using TRIzol reagent (Invitrogen). The cDNAs were synthesized as PrimeScript RT reagents Kit (TaKaRa) manufacturer's instructions. Quantitative real-time polymerase chain reaction (qRT-PCR) was performed in triplicate with LightCycler2.0 (Roche Diagnostics Company) and normalized to β-actin as endogenous control. Endogenous mRNA levels of Sufu, Gli1, MGMT and β-actin were determined with SYBR PrimeScript RT-PCR Kit (TaKaRa). The PCR primers designed and synthesized by Sangon Biotech (Shanghai) were listed in Supplementary Table S3. The relative quantitation value for each target gene was expressed as 2^−ΔΔCt^ as previous described [48].

### Western blot, immunofluorescence, immunoprecipitation and immunohistochemistry

Western Blot (WB), immunofluorescence (IF) and immunohistochemistry (IHC) assays were performed as previously described [49, 50]. Rabbit anti-Sufu (1:1000, Cell Signaling Technology), rabbit anti-Gli1 (1:1000, Cell Signaling Technology), rabbit anti-fibrillarin (1:1000, Proteintech) mouse anti-β-actin, anti-GAPDH antibody (1:1000, Sigma-Aldridge) and HRP-labeled secondary antibody (1:4000, Zsbio) were used in WB. Rabbit antibodies against Sufu, Gli1 (1:100, Bioss) and TRITIC labeled secondary antibody (1:100, Zsbio) were used for IHC and IF.

For co-Immunoprecipitation (co-IP), the cells were lysed using RIPA buffer and incubated with 20 μL of protein-A/G PLUS-Agarose beads (Santa Cruz) and 1 μg of the appropriate primary antibodies at 4°C overnight. After washing 3 times with RIPA, the samples were analyzed through WB.

### Gli reporter assay

To determine Gli activity, a reporter was kindly provided by Gregory J. Gores, M.D. (Division of liver pathobiology, Mayo Clinic, Rochester, MN) containing eight directly repeated copies of a consensus Gli binding site (8x-Gli) downstream of the luciferase gene was used. The procedure of assay was illustrated previously [50]. Briefly, 24 h after transfection, cells were treated with different intervene for another 24 h. Then, cell lysates were prepared and quantified using the Dual-Luciferase Reporter Assay System (Promega). Data were expressed as fold increase over control.

### Tumor xenograft study

In brief, Sufu-overexpressed U87 cells and control U87 cells (3 × 10^5^ cells per mouse in 3 μL) transfected with luciferase lentivirus were injected into the intracranial of 5-week-old female nude mice as described earlier [50]. Each group has 7 mice. After 20 days, tumors were measured by bioluminescence using an IVIS Lumina Imaging System (Xenogen). After 45 days, left mice were sacrificed. Paraffin sections (4 mm) were stained with hematoxylin and eosin and used for IHC.

### Statistical analysis

The significance of differences between two groups was estimated with the Student t test and Chi-square test. Overall survival (OS) curves were plotted according to the Kaplan–Meier method, with the log-rank test applied for comparison. Pearson or Spearman correlationship analyses were used to confirm relationship between different genes. Cox regression was used to correlate Sufu expression with OS of high-grade glioma patients. All differences were considered statistically significant at the level of *P* < 0.05. Statistics were performed using the SPSS Graduate Pack 19.0 statistical software (SPSS, Chicago, IL, USA).

### Databases used

Chinese Glioma Genome Atlas (CGGA) database (http://www.cgga.org.cn); the Cancer Genome Atlas (TCGA) database (http://cancergenome.nih.gov); the Repository for Molecular Brain Neoplasia Data (REMBRANDT, http://caintegratorinfo.ci.nih.gov/rembrandt); GSE16011 data (http://www.ncbi.nlm.nih.gov/geo/query/acc.cgi?acc=GSE16011).

## SUPPLEMENTARY FIGURES AND TABLES


