# Supplementary material for: Suppressor of fused (Sufu) represses Gli1 transcription and nuclear accumulation, inhibits glioma cell proliferation, invasion and vasculogenic mimicry, improving glioma chemo-sensitivity and prognosis
Source: Oncotarget. 2014 Oct 29;5(22):11681–94. doi: 10.18632/oncotarget.2585 (PMC4294353; doi:10.18632/oncotarget.2585)
Supplement: Supplementary file 1 [file oncotarget-05-11681-s001.pdf]

SUPPLEMENTARY FIGURES AND TABLES

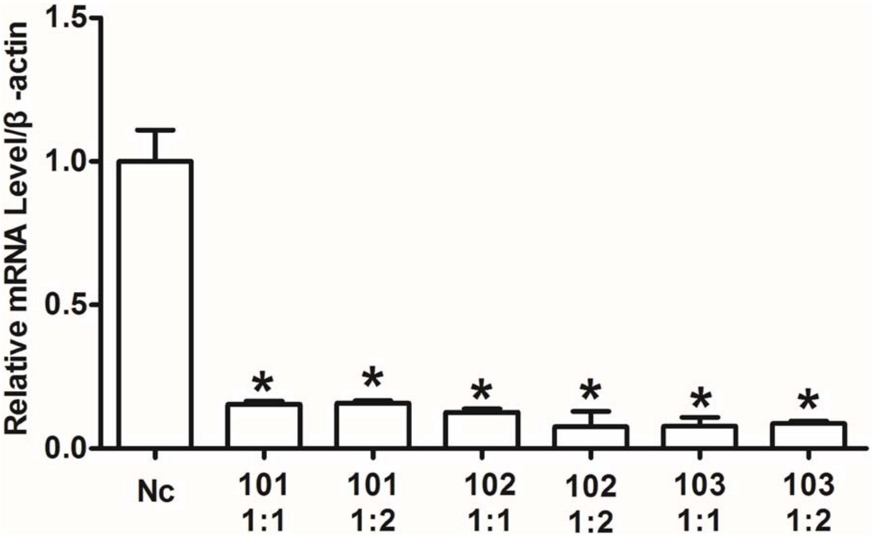

Supplementary Figure S1: Knockdown efficiency of siRNA. Data represent mean±SEM of three replicates. *$P < 0.05$.

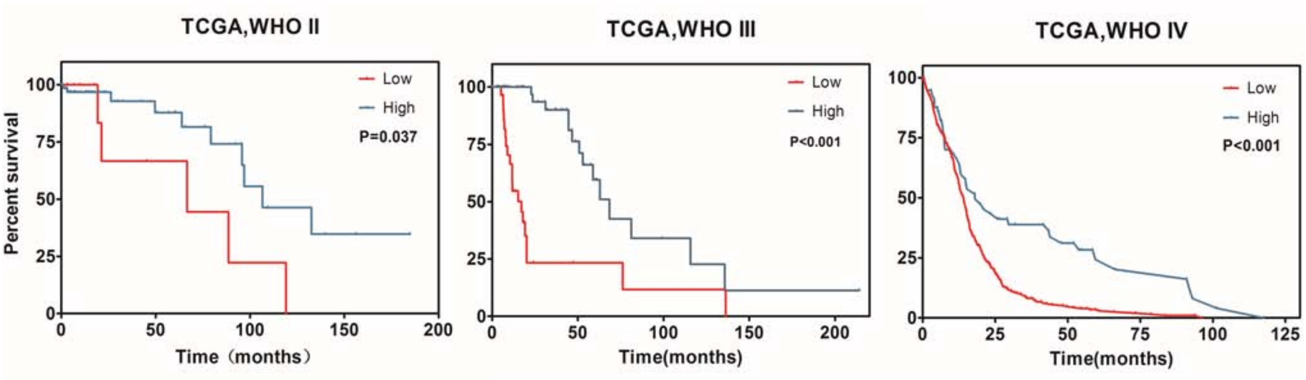

Supplementary Figure S2: The prognostic value of Sufu in glioma tissues of TCGA. According to Sufu expression level, patients with every grade could be divided into two groups with significantly different prognosis.

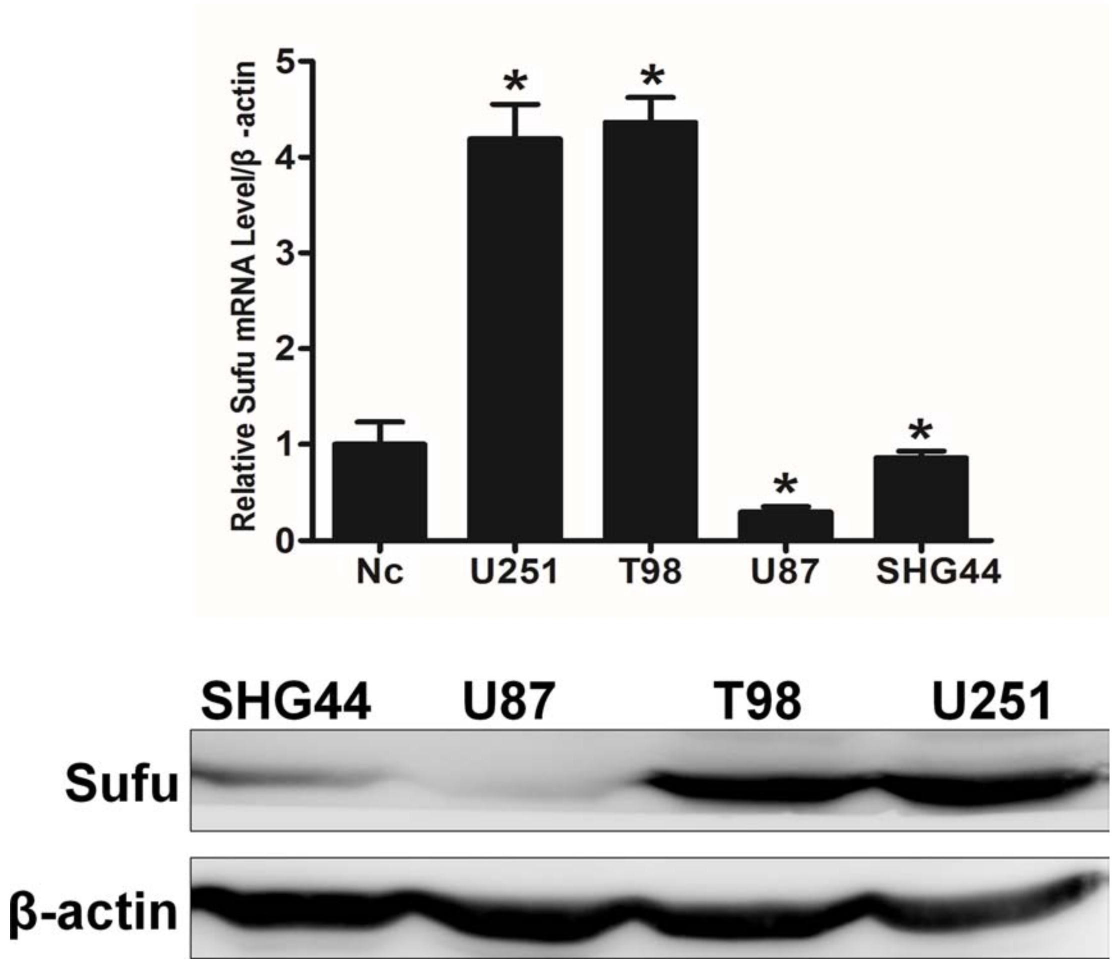

Supplementary Figure S3: mRNA and protein expression of Sufu in different human glioma cell lines. A tumor tissue obtain from glioblastoma patient was set as negative control. Data represent mean±SEM of three replicates. *$P < 0.05$.

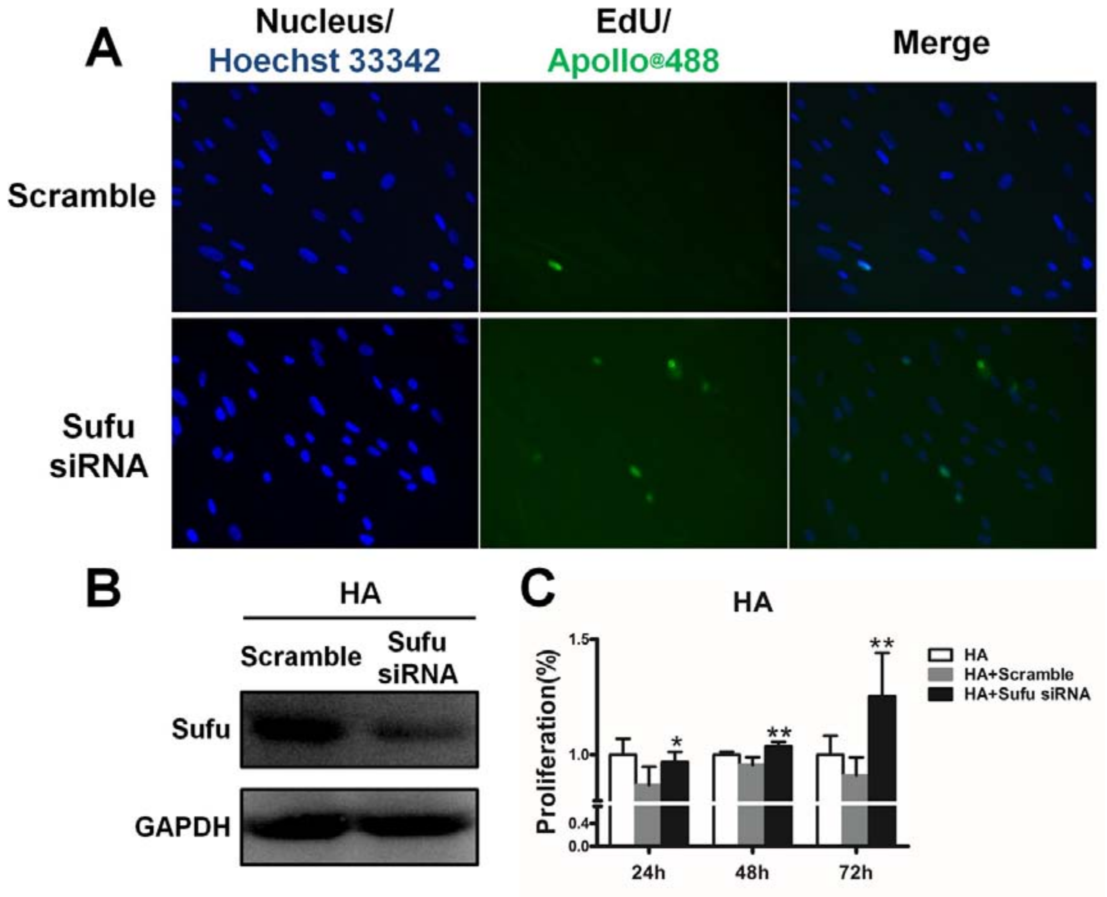

Supplementary Figure S4: Knockdown of Sufu suppressed Human Astrocyte cells proliferation. (A) EdU proliferation assay. HA cells of negative control group and Sufu siRNA group incubated with EdU overnight. Nuclear (Hoechst 33342, blue) and EdU (Apollo@488, green) were stained. **(B)** Sufu siRNA efficiency was tested by WB assay. **(C)** HA cells viability was tested by MTT assay. Data represent mean±SEM of three replicates. $^{*}P < 0.05$; $^{**} P < 0.01$.

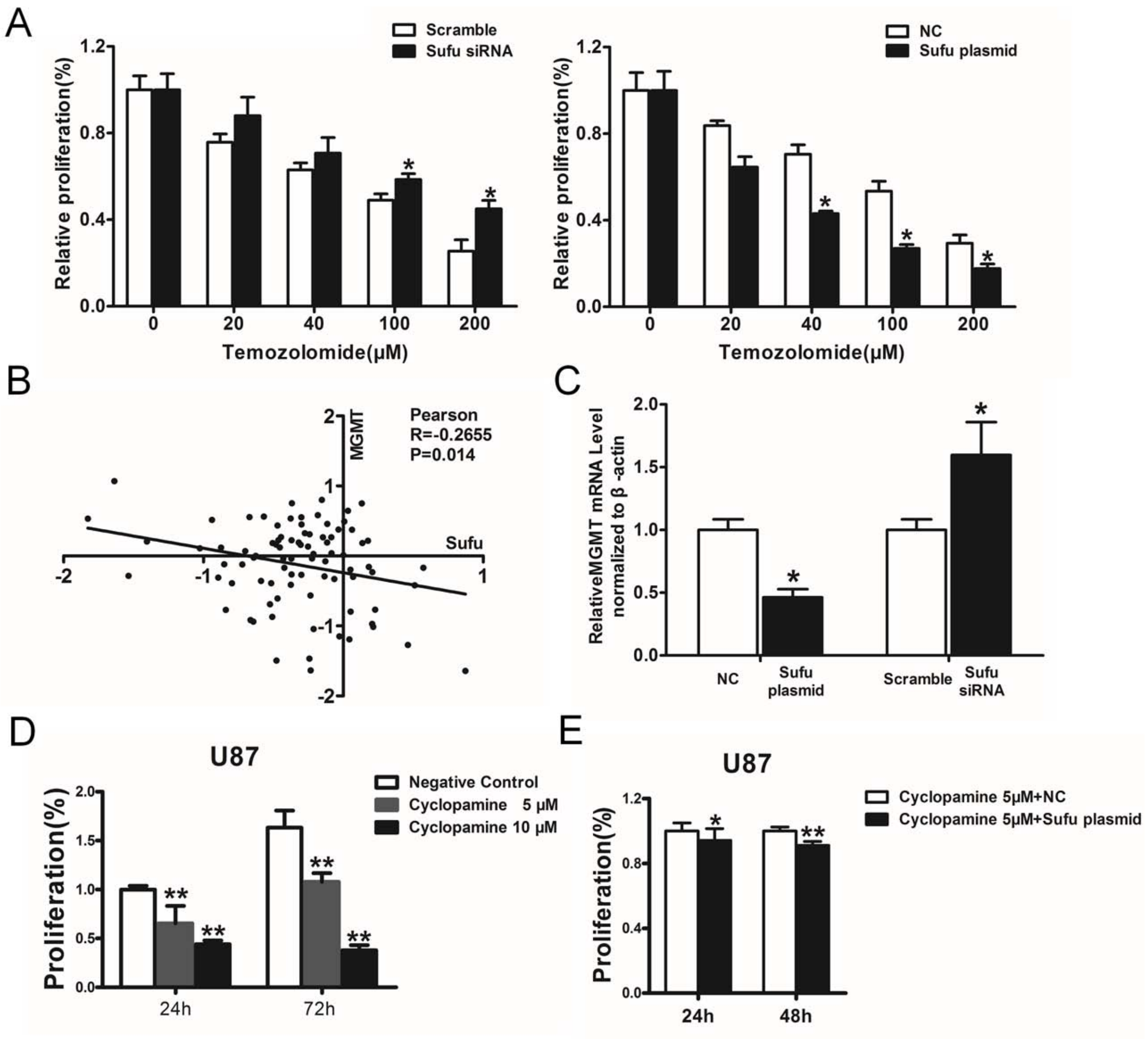

Supplementary Figure S5: Sufu enhanced glioma cells sensitivity to Temozolomide and Cyclopamine. (A) Transfected U87 cells viability exposed to various TMZ concentrations ranged from 0 to 100µm. **(B)** Pearson correlationship of Sufu and MGMT expression of CGGA data. **(C)** MGMT mRNA expression changes after transfection in U87 cells. **(D-E)** U87 cells viability after exposed to 5 µM/10 µM Cyclopamine as well as combined therapy of Sufu plasmid and 5 µM were tested by MTT assay. Data represent mean±SEM of three replicates. *$P < 0.05$; **$P < 0.01$.

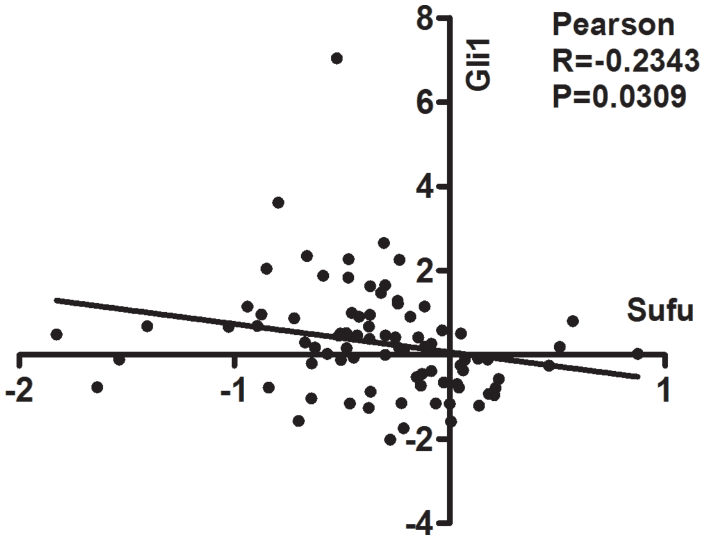

Supplementary Figure S6: Pearson's correlation analysis of Sufu and Gli1 in CGGA database. R= −0.2343, *P* = 0.0309.

Supplementary Table S1. Clinical pathologic parameters of 30 glioma samples from Department of Neurosurgery of 2nd Affiliated Hospital of Harbin Medical.

No.	Age	Sex	Pathology	No.	Age	Sex	Pathology
1	31	Female	Astrocytoma II	16	8	Male	Anaplastic Astrocytoma III
2	13	Female	Pilocytic Astrocytoma I	17	45	Female	Anaplastic Astrocytoma III
3	33	Male	Pilocytic Astrocytoma I	18	42	Female	Anaplastic Astrocytoma III
4	45	Female	Astrocytoma II	19	59	Male	Anaplastic Astrocytoma III
5	51	Male	Oligoastrocytoma II	20	20	Male	Anaplastic Astrocytoma III
6	52	Male	Astrocytoma II	21	31	Male	Anaplastic Astrocytoma III
7	9	Male	Astrocytoma II	22	63	Female	Anaplastic Oligodendroglioma III
8	28	Female	Oligodendroglioma II	23	42	Male	Glioblastoma IV
9	45	Male	Astrocytoma II	24	6	Male	Glioblastoma IV
10	50	Male	Astrocytoma II	25	58	Female	Glioblastoma IV
11	43	Male	Astrocytoma II	26	49	Female	Glioblastoma IV
12	51	Male	Oligoastrocytoma II	27	49	Male	Glioblastoma IV
13	63	Male	Anaplastic Astrocytoma III	28	48	Female	Glioblastoma IV
14	32	Male	Anaplastic Astrocytoma III	29	59	Male	Glioblastoma IV
15	52	Female	Anaplastic Astrocytoma III	30	50	Female	Glioblastoma IV

Supplementary Table S2. The sequence of Sufu siRNA and its target sequence.

Product	Target Sequence	Sequence
Si-h-Sufu_101	CGGCCTGAGTGATCTCTAT	5′ CGGCCUGAGUGAUCUCUAUdTdT 3′<br>3′ dTdTGCCGGACUCACUAGAGAUA 5′
Si-h-Sufu_102	GATCCACACCTGCAAGAGA	5′ GAUCCACACCUGCAAGAGAdTdT 3′<br>3′ dTdTCUAGGUGUGGACGUUCUCU 5′
Si-h-Sufu_103	GCAGCTTGAGAGCGTACAT	5′ GCAGCUUGAGAGCGUACAUdTdT 3′<br>3′ dTdTCGUCGAACUCUCGCAUGUA 5′

Supplementary Table S3. The sequence of real-time PCR primers.

Gene	Forward Sequence	Reverse Sequence
β-actin	CTGGGACGACATGGAGAAAA	AAGGAAGGCTGGAAGAGTGC
Sufu	CCAGACCCCTTGGACTATGTTA	GACTCCCCAGTTTCTCTCTTCA
Gli1	ATCCTTACCTCCCAACCTCTGT	AACTTCTGGCTCTTCCTGTAGC
MGMT	CCTGGCTGAATGCCTATTTC	TGTCTGGTGAACGACTCTTGC
